# Alpha-Pinene-encapsulated lipid nanoparticles diminished inflammatory responses in THP-1 cells and imiquimod-induced psoriasis-like skin injury and splenomegaly in mice

**DOI:** 10.3389/fimmu.2024.1390589

**Published:** 2024-10-29

**Authors:** Tao-Yu Li, Wan-Li Liang, Yi-Ming Zhao, Wan-Dong Chen, Hong-Xia Zhu, Yuan-Yuan Duan, Han-Bo Zou, Sha-Sha Huang, Xiao-Jun Li, Wei Kevin Zhang

**Affiliations:** ^1^ School of Pharmaceutical Sciences, Guangzhou Medical University, Guangzhou, Guangdong, China; ^2^ Guangzhou National Laboratory, Guangzhou International Bio Island, Guangzhou, Guangdong, China; ^3^ School of Pharmacy, Faculty of Medicine, Macau University of Science and Technology, Macao, Macao SAR, China; ^4^ School of Life Sciences, Sun Yat-sen University, Guangzhou, China; ^5^ Key Laboratory of Viral Pathogenesis and Infection Prevention and Control (Jinan University), Ministry of Education, Guangzhou, China; ^6^ School of Pharmaceutical Sciences, South-Central University for Nationalities, Wuhan, China

**Keywords:** nanoliposomes, α-pinene, psoriasis, cytokines, NF-κB signaling

## Abstract

**Introduction:**

Psoriasis, a persistent skin condition caused by the disorder of the immune system, impacts approximately 1.25 million individuals globally. Nevertheless, the presence of adverse effects in conventional clinical drugs necessitates further exploration of novel medications or combination therapies to mitigate these reactions and enhance their effectiveness.

**Methods:**

Hence, our intention here in this paper is to utilize the lipid nanoparticle delivery system for overcoming the volatility and hydrophobic properties of α-pinene, a naturally occurring compound renowned for its anti-inflammatory and antiviral effects, and further explore its potential pharmacological applications both *in vitro* and *in vivo*.

**Results:**

The production of α-pinene lipid nanoparticles (APLNs) was achieved through the utilization of high pressure homogenization methods. APLNs was successfully fabricated with enhanced stability and water solubility. Meanwhile, the application of APLNs could drastically reduce the expression of lipopolysaccharide (LPS)-induced inflammation-related factors in THP-1 cells. Administration of APLNs to a mouse model of auricular swelling could effectively reduce redness and swelling in the auricles of mice as well. Furthermore, APLNs were also found to alleviate skin damage in mice with Imiquimod (IMQ)-induced psoriasis model, as well as decrease the levels of psoriasis-related protein nuclear factor kappa-B (NF-κB) and interleukin-17 (IL-17), interleukin-23 (IL-23), and other inflammation-related cytokines. More importantly, utilization of APLNs successfully mitigated the systemic inflammatory reactions in mice, resulting in the reduction of spleen-to-body ratio (wt%) and of inflammatory cytokines’ expression in the serum.

**Discussion:**

Overall, our results suggest that with the help of lipid nanoparticle encapsulation, APLNs possess a better pharmacological effect in anti-inflammation and could potentially serve as an anti-psoriasis drug.

## 1 Introduction

Nowadays, drug discovery is still, to a large extent, relies on natural compounds (1, 2). Many conifer-derived essential oils contain α-pinene as their primary or secondary metabolite (3), which is known for its volatile and hydrophobic characteristics, as well as its fresh pine aroma and woody taste (4). Extensive research has already been conducted on the biological functions of α-pinene, encompassing effects of anti-microbes, inflammation reduction, reactive oxygen species (ROS) clearance, cell protection, tumor elimination, etc (5–9). Application of α-pinene could reduce the inflammatory reactions in mouse peritoneal macrophages treated with lipopolysaccharide via the down-regulation of mitogen-activated protein kinases (MAPKs) and nuclear factor kappa-B (NF-κB) (10). Additionally, α-pinene have also been shown to suppress the activation of inflammatory mediators including NF-κB, tumor necrosis factor α (TNF-α), and interleukin-6 (IL-6) in HaCat cells under ultraviolet-A (UVA) challenge, along with the declines in ROS production, lipid oxidation, and DNA damage (11, 12). However, the volatility and hydrophobic nature of α-pinene (soluble in some organic solvents but not aqueous condition) seriously restrict the prevalence of its clinical use.

Lipid nanoparticles (LNs), on the other hand, are powerful delivery tools, and have been utilized in a variety of industries, including pharmaceuticals, cosmetics, therapeutic imaging, nourishment, and other innovative fields (13). It consists of solid lipids or mixtures of solid and liquid-crystalline lipids, with particle sizes ranging from 40 to 1000 nm (13, 14). Due to the advantages of LNs, many drugs packaged in LNs have promising application prospects: High levels of bio-compatibility and biodegradability, thus leading to high levels of safety; Simple of use for industry manufacture; Encapsulation of imbed content, thus maintaining long-term stability and prolonged drug release; Tiny size facilitating the internalization of imbed content; Surface modifiable and thus promote drug targeting; and so on (15). Meanwhile, in our previous study, sesame oil has been perfectly encapsulated in LNs and they together induced apoptosis in mouse macrophages (RAW264.7) through a mitochondria-dependent pathway (16). This inspires us that LNs might be a wonderful tool for enhancing the water solubility and aqueous stability of α-Pinene, which would be beneficial to its clinical application, making full use of its anti-inflammatory activities in particular.

The presence of noxious stimuli including microbe infection and tissue injury, could trigger a direct response referred to as inflammation (17). The prevailing belief is that a controlled inflammatory reaction yields advantage such as safeguarding against infections, yet it could be detrimental when dysregulated and result in symptoms such as septic shock (17). When macrophages come into contact with pathogen-associated molecular patterns (PAMPs) such as lipopolysaccharide (LPS), which are the building blocks of the outer membranes of gram-negative bacteria, they activate downstream signaling pathways like MAPK and NF-κB, resulting in the production of various molecules including cytokines, chemokines, and ROS (18).

In addition, psoriasis and inflammation share significant commonalities in their mechanisms. Psoriasis, a skin disorder characterized by its long-lasting and recurring nature, is caused by the immune-mediated manifests in the skin and/or joints (19, 20). Approximately 1 ~ 2% of the global population is believed to be affected by psoriasis (21). Topical therapies alone can effectively manage mild psoriasis in most of patients (22). It is believed that psoriasis is a multifaceted ailment, stemming from the interaction of genetic predisposition and environmental factors (23). It is evident that both adaptive and innate immunity pathways play roles in the pathogenesis of psoriasis (24), these genetically susceptible patients are stimulated by external stimuli (e.g., UV or systemic drugs) production of interleukin-23 (IL-23), leading to the induction of various cytokines (including IL-17) that subsequently modify the function of keratinocytes and other skin tissue cells, either directly or through secondary mediators (23, 25). NF-κB, made up of p65 and p50 proteins, has the capacity to regulate the expression of a variety of genes that are enrolled in the cellular response to external stimuli (26), thus promoting the production of interleukin-1β (IL-1β), interleukin-4 (IL-4), Interferon-gamma (IFN-γ), and other essential pro-inflammatory factors, resulting in the emergence of psoriasis. Furthermore, several cytokines such as IFN-γ, interleukin-12 (IL-12), interleukin-22 (IL-22) and IL-23, which are regulated by the Janus Kinase- Signal transducers and activators of transcription (JAK-STAT) signaling pathway, have been identified as having a role in the development of psoriasis (27). Nowadays, treatments for mild psoriasis mainly include topical corticosteroids, vitamin D analogues, etc (28). However, the side effects of corticosteroids, particularly when used over a period of time in child, may be the inhibition of the functioning of the hypothalamic pituitary and hence influence on the adrenal axes, whereas the latter vitamin D analogues could cause skin irritation, burning, itching, and edema (28–32). The limited availability of medications for psoriasis sufferers due to these side effects necessitates the exploration of novel drugs. Hence, in the current study, we utilized the imiquimod-induced mice model of psoriasis to explore the potential therapeutic effects of APLNs as well.

## 2 Materials and methods

### 2.1 Fabrication and characterization of APLNs

The APLNs were prepared using the hot homogenization technique, as shown in the figure. To put it succinctly, the oil phase was made up of 240 mg of egg yolk lecithin and 7.52 ml of α-pinene, and then heated to 60°C before being evenly blended. Likewise, the water phase consisted of 30.7 ml of ultrapure water, 0.9 ml of glycerol, and 0.4 ml of Tween-80, all of which were preheated to 60°C and thoroughly combined, pertinent vendors of reagents and their catalog numbers are presented **Supplementary Table S1**. Subsequently, the oil phase was gradually incorporated into the water phase and agitated for a duration of 10 minutes. The PT-2500E adjustable high-speed homogenizer was used to create the initial emulsion after 10 minutes of high-speed shearing at 10000 rpm. The mixture was emulsified 20 times under a pressure of 1000 bar using an ATS high-pressure homogenizer (AH-NANO, Canada) to form nano-emulsion. The dispersion, after being prepared, was cooled to room temperature and then sterilized with 0.22 μm sterile syringe filters. The obtained APLNs was stored at 4°C away from light for further experiments.

### 2.2 Measurement of particle size and zeta potential of APLNs

The measurement of particle size and zeta potential was conducted using the Zetasizer Nano series (MAL1251109, Malvern, UK). 2 μl APLNs was dissolved in 1 ml ultrapure water and put it into the instrument for detection. All measurements are presented as summaries of triplicates.

### 2.3 Cell culture and electron microscope observation

Human monocyte leukemia cells (THP-1, iCell-h213) were purchased from the iCellbioscience inc (Shanghai, China) and were cultured in a CO2 incubator according to the supplier’s instructions. 1×10^4^ cells were cultured into a well of 96-well plate and incubated with RPMI 1640 containing 10% FBS, added to different concentrations APLNs for 24 h and electron microscope observation.

### 2.4 RNA-sequencing and analysis

RNA samples were collected and the Illumina TruseqTM RNA sample prep Kit was utilized for the creation of cDNA libraries. Sequence-by-synthesis single reads of 54-base-length using the Hiseq2000 Truseq SBS Kit (v3-HS, Illumina) were generated on the HiSeq X system. Raw data were collected and count matrix were processed with a fastQC-Trimgalore-Hisat2-FeatureCounts pipeline in Linux. R were used for the calculation of TPM matrix and follow-up DEG analysis via the DESeq2 package, with the criteria as follows: p_adj_ < 0.05 & |log_2_FC| > 1.5. Gene enrichment analysis were performed in the Metascape website (metascape.org) Nature Commun. 2019 10(1):1523 (33).

### 2.5 Quantitative PCR

A total of 1×10^6^ cells were seeded into 35 mm culture dish and added to 1 μg/ml LPS for 2 h to incubate. Following the exposure to various concentrations of APLNs, cells were cultured in a sequential manner for a duration of 24 h. Total RNA was extracted from THP-1 cells treated as indicated using RNAiso Plus reagent (AL42059A, Takara), after reversely transcribed with a PrimeScriptRT reagent kit (RR047A, Takara) and under 37°C, 15 min; 85°C, 5 s; 4°C, 5 min conditions with the gene amplification instrument (PCR, Trident960, Heal Force). The mRNA levels were analyzed by qPCR using CFX96 Touch PCR System (Bio-Rad, Singapore). All qPCR reactions were performed in these situations: an initial denaturation step at 95°C for 30 s, followed by 40 cycles of denaturation at 95°C for 15 s, annealing and extension at 60°C for 60 s. PCR reactions were carried out in triplicates. GAPDH was used as an internal control. The relative amount of each cDNA was determined using the 2-ΔΔCt method. The primer sets used in THP-1 cells can be found in **Supplementary Table S2**.

### 2.6 Western blot analysis

Total proteins of THP-1 cells were extracted with RIPA buffer, and their concentration was measured with a BCA protein quantification kit. Protein samples were used for SDS gel electrophoresis (6%, 10%, 12.5% SDS gels), and proteins were transferred to PVDF membranes. The membranes were blocked with 5% milk powder solution (TBST; Tris-buffered saline, 0.1% v/v Tween-20) for 1 h and incubated with different primary antibodies overnight at 4°C. On the subsequent day, following the elimination of the primary antibody, the membranes underwent three washes with TBST for a duration of 5 minutes, followed by a 1 h exposure to the secondary antibody (Rabbit) at ambient temperature. Finally, the membranes underwent another round of washing and were captured through the utilization of Clarity Western ECL developer and an integrated imaging analysis system (SmartChemi 910 PLUS, China), **Supplementary Table S1** elaborates on the pertinent antibodies and reagents, specifying the vendors, their product catalog numbers, and dilution ratios. The ImageJ software was used to measure the protein bands, and the Instat software was used to analyze them (GraphPad Prism 7, La Jolla, USA).

### 2.7 Animal care

This study followed the Guide to Animal Experimentation in regard to the care and utilization of animals and the implementation of experimental protocols, Ruiye model animal (Guangzhou) Biotechnology Co., Ltd and the Committee of Research Facilities for Laboratory Animal Sciences, Ruiye model animal (Guangzhou) Biotechnology Co., Ltd, China. The protocols were approved by the Committee on the Ethics of Animal Experiments of the Ruiye model animal (Guangzhou) Biotechnology Co., Ltd, China (Permit number: RYEth-20220317286).

### 2.8 Mice ear swelling *in vitro* experiments with APLNs

Prior to the experiment, thirty male Kunming mice (8 weeks old, 20–22 g) were acclimated to their environment for a duration of 7 days, ensuring they were free from specific pathogens. The mice were nurtured in a 12 h light-dark cyclic light setting for a duration of 7 days, while being provided with a basal diet within a temperature range (22–25°C) in the mice room. The mice in this animal experiment were divided into six groups in a random manner: the control group (transdermal drug of the right ear with normal saline 50 μl), the APLNs group (transdermal drug of the right ear with APLNs 50 μl), the LNs group (transdermal drug of the right ear with LNs 50 μl), the Xylene group (ear inflammatory model of the right ear with Xylene 50 μl and transdermal drug of the right ear with APLNs 50 μl), the Xylene + LNs group (ear inflammatory model of the right ear with Xylene 50 μl and transdermal drug of the right ear with LNs 50 μl). At the beginning of the experiment, the right ear of each group of mice was given the corresponding drug through transdermal administration. After 5 minutes, each group of mice was smeared with xylene to induce swelling of the auricle. The mouse auricles were observed and measured prior to percutaneous administration, 30 minutes following the administration of xylene, 60 minutes after the application of xylene, and 120 minutes after the application of xylene. Ultimately, the mouse auricle tissue was collected for subsequent experimentation.

### 2.9 Mice back skin for imiquimod-induced psoriasis *in vivo* experiments with APLNs

Thirty-five male BALB/c mice (8 weeks old, 20–22 g) were adapted to the surroundings for 7 days under specific-pathogen free conditions before experiment. All mice were raised in 12 h light–dark cyclic light environment for 7 days and kept on a basal diet in a temperature range (22–25°C) mice room. In this animal experiment, the mice were divided into seven groups in a random manner: the control group (transdermal drug with normal saline), the saline groups (IMQ-induced and transdermal drug with normal saline), the LNs groups (IMQ-induced and transdermal drug with LNs), the APLNs groups (IMQ-induced and transdermal drug with APLNs), the α-pinene (AP) groups (IMQ-induced and transdermal drug with AP), the Calcipotriol-Betamethasone (Cal/Bms) groups (IMQ-induced and transdermal drug with Cal/Bms), and the Fluocinonide (FA) groups (IMQ-induced and transdermal drug with FA). The day prior to the experiment, the mice’s backs were treated with depilatory creams, with a depilation area of 4 cm^2^. The mice’s back skin was smeared with IMQ 0.0625 g from the initial day until the thirteenth day, resulting in the development of psoriasis. The corresponding medications were administered between the fourteen and twenty-two days. Eventually, the back skin, blood and spleen were taken for the next experiment.

### 2.10 Histopathological analysis

Samples of fresh auricle, skin, and spleen tissue were fixed in 10% neutral formalin, embedded in paraffin, cut into 2 μm sections, and stained with hematoxylin and eosin (H&E). Pathological sections were observed by electron microscope (Motic, BA410).

### 2.11 Immunohistochemical staining

The skin tissues were freshly collected and preserved in a 10% neutral formalin solution for histological examination. Paraffin was used to encase the skin tissues, and the paraffin sections measuring 3 μm were subjected to staining with hematoxylin or incubation with antibodies targeting NF-κB, COX-2, IL-6, IL-17A, IL-23, and TNF-α. Images were taken using a microscope.

### 2.12 Multi-analyte flow assay

Allow the blood to clot for at least 30 minutes and centrifuge for 20 min at 1000 x *g*. LEGENDplex™ Multi-Analyte Flow Assay Kit (7410, San Diego, CA) was customized by BioLegend, Inc. Performing the Assay Using a Filter Plate and Vortex mixed beads bottle. By adding 1X Wash Buffer, detection antibodies, 1X Wash Buffer again to each well, resuspend the beads on a plate shaker. The flow cytometer measured samples of inflammation-related cytokines, including IFN-γ and TNF-α, etc.

## 3 Results

### 3.1 Fabrication and characterization of APLNs

**Figure 1:**
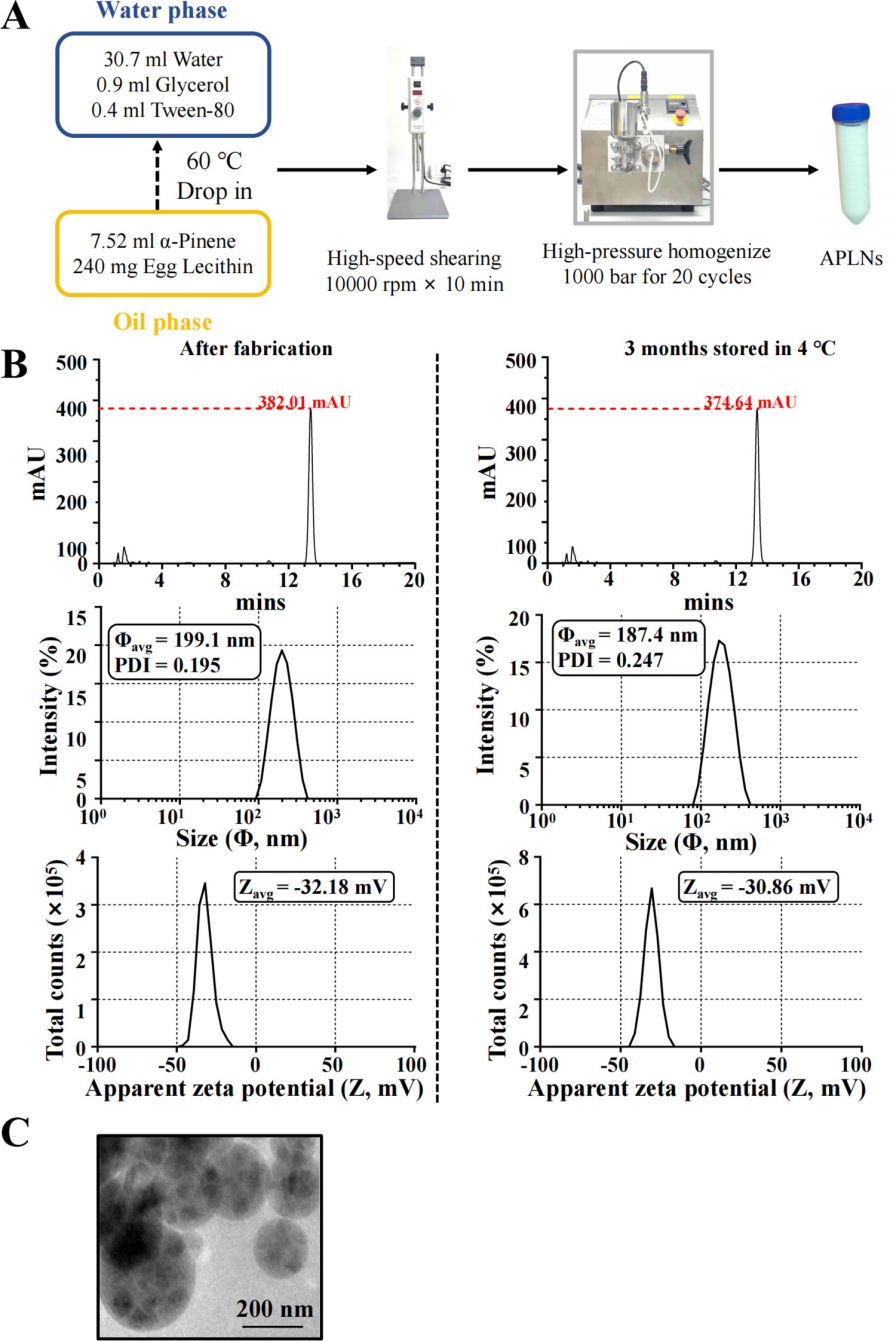
The fabrication and characterization of APLNs. **(A)** The fabrication formula and procedure for APLNs. **(B)** The average size (in diameter), PDI indices, zeta potential, and HPLC-DAD detection, of APLNs immediately after the fabrication or 3 months later (stored in 4°C). **(C)** The TEM images of APLNs, Scale bar 200 nm.

After continuous optimization, the detailed fabrication process of APLNs were shown in **Figure 1A**. By HPLC-DAD detection, the retention times of APLNs after fabrication and storage at 4°C for 3 months were *t*_R_ = 13.39 min and *t*_R_ = 13.35 min, and the peaks were 382.01 mAU and 374.64 mAU, respectively, indicating that the relative content of α-pinene encapsulated by lipid nanoparticles was not change after three months, as shown in **Figure 1B**. **Figure 1B** displays the size and apparent zeta potential of APLNs following their fabrication and storage at a temperature of 4°C for a duration of 3 months. The average size and zeta potential, approximately 200 nm, exhibited minimal variation after fabrication and after 3 months. Particles with a zeta potential lower than -30 mV or higher than +30 mV demonstrate greater stability (34). The zeta potential was all below -30 mV, demonstrating the relative stability of our APLNs, as shown in **Figure 1B**. A polydispersity index (PDI) below 0.3 for liposome and nanoliposome formulations is deemed satisfactory, signifying a homogenous population of phospholipid vesicles (35). **Figure 1B** illustrates the PDI of APLNs after fabrication and after 3 months, which ranged from 0.195 to 0.247, all of which was lower than 0.3, as indicated by the monitoring results. TEM images results further showed that APLNs had a spherical shape, and the spherical periphery was similar to the lipid membrane-like structure, with the dispersed oily phase wrapped in the lipid nanoparticles as depicted in **Figure 1C**. Similarly, TEM images results also showed that the particle size of APLNs was around 200 nm, which was consistent with the above detection results.

### 3.2 LPS-induced THP-1 cells by administering APLNs to modulation of NF-κB, ERK, and NRF2 signaling pathways

After stable APLNs were prepared, we explored the anti-inflammatory molecular mechanism of α-pinene at the cellular level because α-pinene has been reported to have anti-inflammatory effects (10).

**Figure 2:**
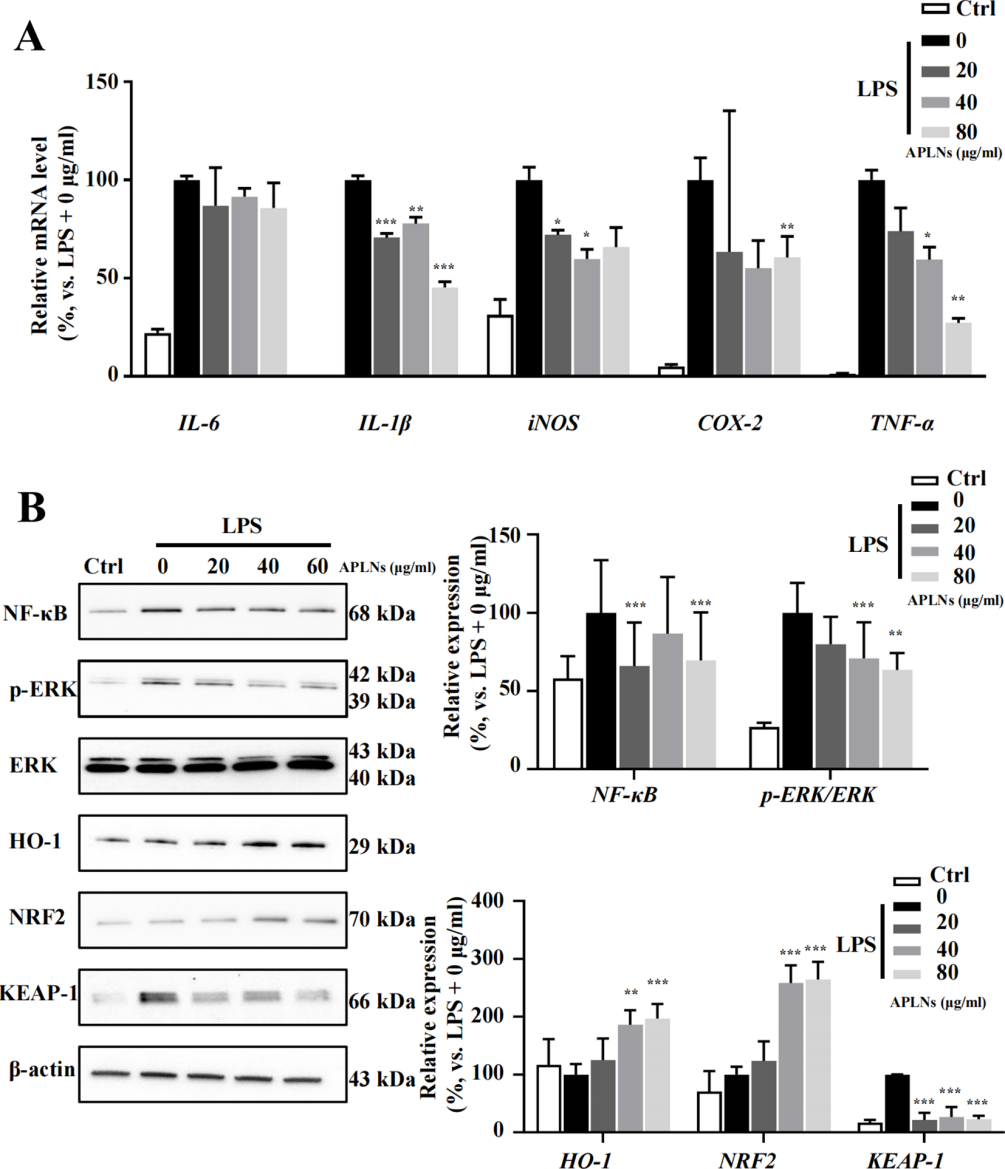
Effects of APLNs on THP-1 cells treated with LPS, including the modulation of NF-κB pathway, oxidative stress-related pathways, and inflammation-related cytokines. **(A)** qPCR results confirmed that iNOS, COX-2, IL-6, IL-1β, and TNF-α were down-regulated dose-dependently by APLNs treatment in LPS-stimulated THP-1 cells. **(B)** Protein expression results in the description that NF-κB, p-ERK/ERK, NRF2, KEAP-1, and HO-1 were modulated by APLNs treatment in LPS-stimulated THP-1 cells. N = 3 independent experiments with duplicates in each. ^*^, ^**^and ^***^, *p* < 0.05, 0.01 and 0.001 respectively, comparing with the LPS + 0 μg/ml group. The molecular weights of detected bands were indicated in the left side of the Western blot.

**Figure 3:**
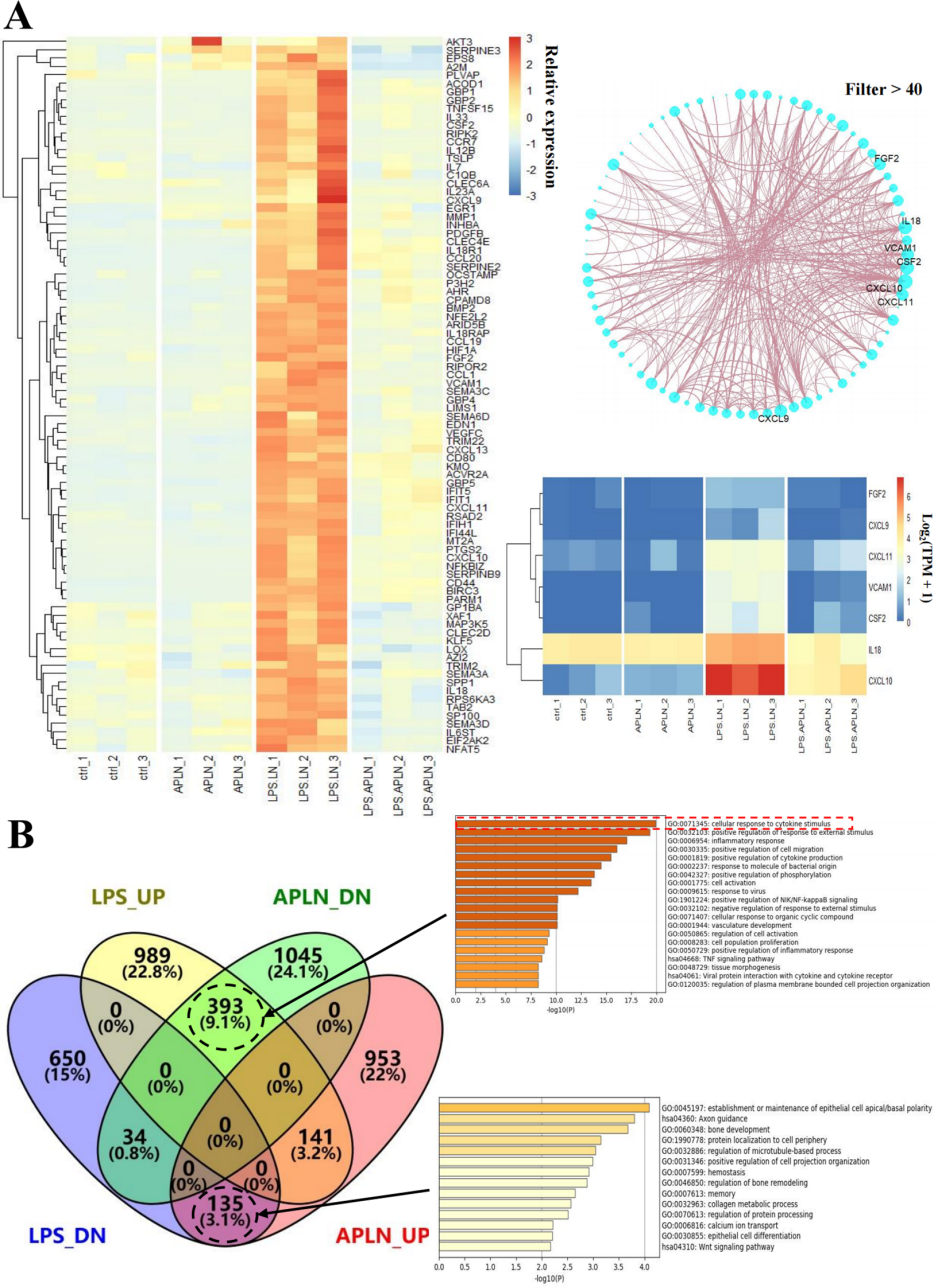
RNA-sequencing and analysis results were used to analyze LPS-induced THP-1 cells’ relative gene expression. **(A)** Immune-related genes, including MAP3K5 (MAPK), NFKBIZ (NF-κB), TNFSF15 (TNF-α), and IL-6, were down-regulated in the LPS + APLNs group compared with the LPS group. **(B)** Serial genetic changes in the cellular response to cytokine stimuli were evident in the LPS group vs LPS + APLNs group.

The quantitative PCR results validated the ability of LPS-induced THP-1 cell administration of APLNs to effectively reduce the levels of inducible nitric oxide synthase (iNOS), cyclooxygenase-2 (COX-2), IL-6, IL-1β, and TNF-α cytokines, as summarized in **Figure 2A**. The disruption of NF-κB, MAPK, or JAK-STAT derived signal pathways is a significant etiologic factor cause of numerous inflammatory, autoimmune, and metabolic disorders (36). Consequently, this article advocates for the impact of APLNs on the aforementioned protein pathways. Western blot results showed that LPS-induced THP-1 cell administration of APLNs was able to effectively suppress the expression of NF-κB and the phosphorylation of Extracellular signal-related kinase (p-ERK), as summarized in **Figure 2B**. Similarly, to better understand the effects of APLNs on LPS-induced THP-1 cells, we performed transcriptome sequencing. The genes in the gene set that were up-regulated and down-regulated were grouped together using DEG analysis and GO/KEGG function enrichment, and both the Ctrl vs LPS and the Ctrl vs LPS + APLNs had a significant amount of up-regulated and down-regulated genes, which were linked to proinflammatory and profibrotic, cytokine activity, and molecule of bacterial activity, as illustrated in **Figure 3B**. Furthermore, **Figure 3A** illustrates that the LPS + APLNs group exhibited a notable decrease in the expression of MAP3K5 (MAPK), NFKBIZ (NF-κB), TNFSF15 (TNF-α), IL-6, and other inflammatory cytokines gene when compared to the LPS group. The cytokine IL-23A, which is typically associated with psoriasis, was found to be down-regulated, thus laying the groundwork for further research into human psoriasis in mice.

Nuclear factor erythroid 2-related factor 2 (NRF2) transcription factor is renowned for its role in controlling the cellular xenobiotic and oxidative stress response (37). Studies have indicated that NRF2 has an influence on inflammation regulation (38). The Western blot analysis confirmed that LPS-induced THP-1 cell administration of APLNs resulted in a substantial decrease in Kelch-like ECH-associated protein 1 (KEAP-1) expression, along with an increase in NRF2 and heme oxygenase-1 (HO-1) expression, suggesting that APLNs may play a role in regulating oxidative stress-related pathways leading to anti-inflammatory effects, as depicted in **Figure 2B**.

Generally, on a cellular basis, APLNs are capable of decreasing the secretion and expression of cytokines associated with inflammation in LPS-induced THP-1 cells. Additionally, APLNs play a role in mitigating inflammation by lowering the levels of NF-κB and p-ERK proteins, thereby regulating signaling pathways. Additionally, they regulate responses to cellular xenobiotic and oxidative stress response through the enhancement of NRF2 and HO-1 protein levels.

### 3.3 LPS-induced THP-1 cells by administering APLNs to modulation other genes

The sequencing results provided insight into the ability of LPS-induced THP-1 cells to increase the expression of genes like FGF2, CXCL9, CXCL11, VCAM1, CSF2, IL-18, and CXCL10, while the aforementioned genes exhibited a notable decrease in expression following the administration of APLNs, as depicted in **Figure 3A**. This implies that our APLNs may also exert regulatory influence on fundamental fibroblast growth factor (bFGF), chemokines, vascular cell adhesion molecule (VCAM), and Granulocyte-macrophage Colony Stimulating Factor (GM-CSF) at the site of inflammation, offering potential avenues for future research.

### 3.4 APLNs relieves xylene-induced ear swelling in mice

**Figure 4:**
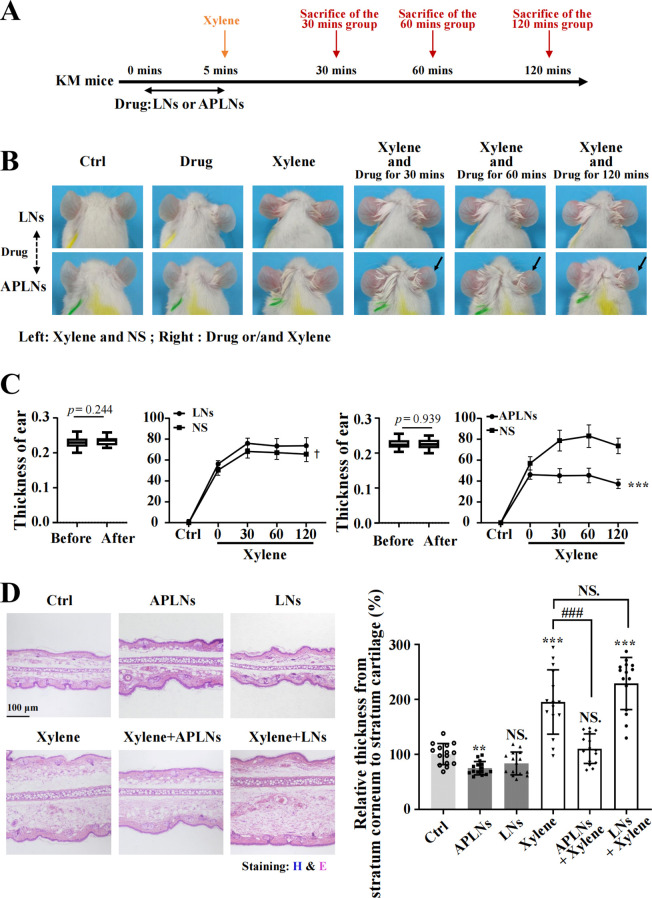
APLNs relieved xylene-induced ear swelling in KM mice. **(A)** Schematic graph of *in vivo* auricular swelling model experiments. **(B)** Xylene-induced mice were treated with normal saline in the left auricle and APLNs in the right ear, and pictures of the mouse auricle were taken at 30 mins, 60 mins, and 120 mins. **(C)** Mouse auricle thickness statistics. The thickness of the treated auricle decreased in the APLNs group while remaining unchanged in the LNs group. N = 5 mice for the all groups. ^***^, *p* < 0.001 comparing with the left auricle. **(D)** H&E staining mouse auricular samples and counted relative thickness from stratum corneum to stratum cartilage. Scale bar 100 μm. N = 5 mice for the all groups. NS., not significant. ^**^ and ^***^ represents *p* < 0.01 and *p* < 0.001 comparing with the Ctrl. ^###^ represents *p* < 0.001 comparing with the Xylene group.

Inquiry at the cellular level above has demonstrated that APLNs have significant anti-inflammatory capacity, followed by anti-inflammatory capacity *in vivo* at the animal level. To enhance the description of its impact on xylene-induced auricular swelling in mice, the corresponding medications were administered to the right auricle of mice, and the mice’s auricle was stimulated by xylene for a duration of 5 minutes. Observed and sacrificed at 30 minutes, 60 minutes, and 120 minutes, as demonstrated in **Figure 4A**. The evolution of mouse auricles is illustrated in **Figure 4B**, highlighting that the APLNs groups experienced less ear swelling and redness compared to the LNs groups at intervals of 30, 60, and 120 minutes. Meanwhile, the results depicted in **Figure 4C** indicated that xylene-induced thinning of the mice’s auricle was observed in APLNs, whereas the thickness of the mouse auricle remained unaltered in LNs. H&E staining further validated the ability of xylene-induced mice auricle administration of APLNs to reduce the relative thickness between the stratum corneum and the cartilage layer, as depicted in **Figure 4D**. The above results indicate that APLNs have the potential to reduce ear swelling in mice induced by xylene and imply their possible anti-inflammatory properties.

### 3.5 APLNs ameliorate IMQ-induced psoriatic skin lesion in mice

**Figure 5:**
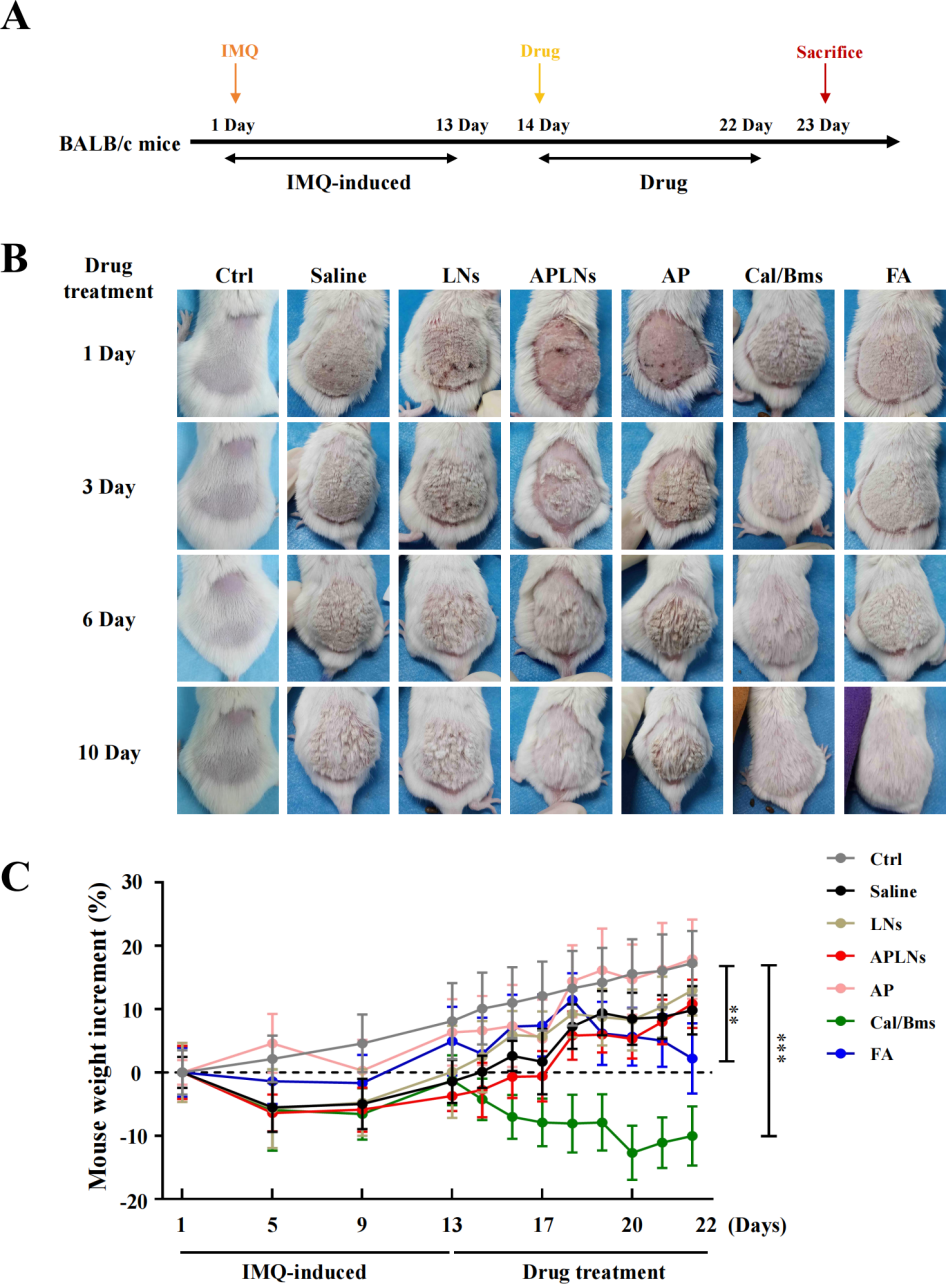
Phenotypic effects of administered APLNs on IMQ-induced human psoriasis in mice. **(A)** Schematic graph of *in vitro* IMQ-induced human psoriasis model experiments. **(B)** Different drugs were administered to IMQ-induced psoriasis of the back skin of mice, and pictures of the back skin were taken on days 1, 3, 6, and 10. **(C)** Changes in body weight after administration of different drugs in each group. N = 5 mice for the all groups. ^**^ and ^***^ represents *p* < 0.01 and *p* < 0.001 comparing with the Ctrl.

**Figure 6:**
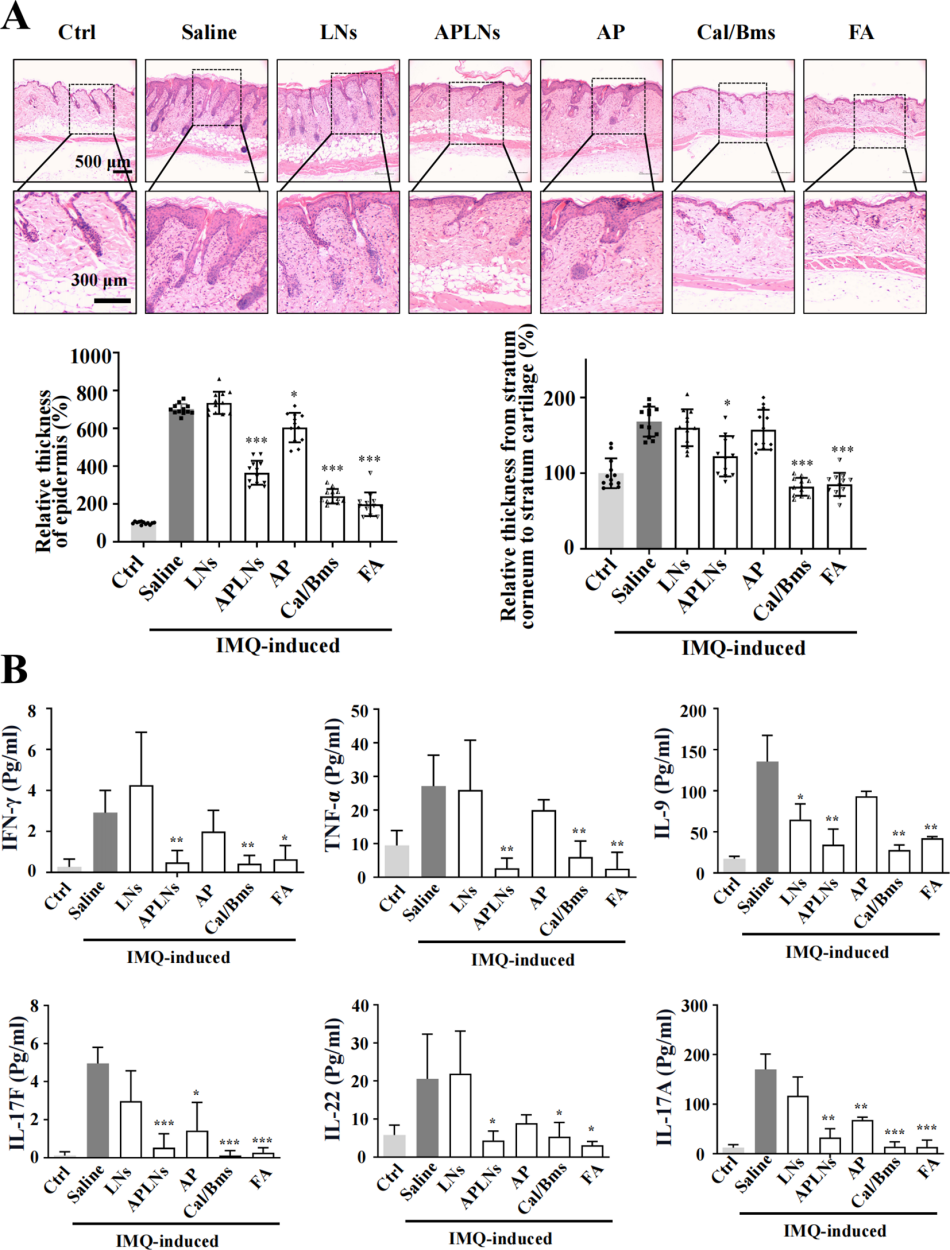
Effects of APLNs on related cytokines in the back and serum on IMQ-Induced psoriasis in mice. **(A)** H&E staining mouse skin and counted the relative thickness of epidermis and the relative thickness from stratum corneum to stratum cartilage. Scale bar, 500 μm for lower magnification and 300 μm for the higher. N = 5 mice for all groups. ^*^ and ^***^ represents *p* < 0.05 and *p* < 0.001 comparing with the Saline. **(B)** The concentration of inflammatory-related cytokines IFN-γ, TNF-α, IL-9, IL-17F, IL-22, and IL-17A in the serum of mice in each group (Pg/ml). N = 5 mice for all groups. ^*^, ^**^ and ^***^ represents *p* < 0.05, *p* < 0.01 and *p* < 0.001 comparing with the Saline.

The above data fully confirmed that APLNs can regulate the molecular mechanism of inflammation and alleviate the redness and swelling of the auricle in mice, which inspired us to determine whether APLNs can affect psoriasis, which overlaps with the molecular mechanism of inflammation (23). We utilized the IMQ-induced psoriasis-like mice model to confirm the therapeutic effect of APLNs on psoriatic skin lesions by APLNs, LNs, α-pinene stock solution, as well as positive drugs. The mice that were exposed to IMQ exhibited typical back inflammation similar to psoriasis, including erythema, scaling, and thickening, in comparison to the control group, as shown in **Figure 5B**. The topical application of APLNs, Cal/Bms, and FA had a profound impact on skin lesions during the course of the treatment, as evidenced by the amelioration in skin inflammation, as illustrated in **Figure 5B**. According to the status of erythema and desquamation on day 8, administration of APLNs, Cal/Bms, and FA significantly reduced the severity of IMQ-induced psoriasis compared with the IMQ model group, as demonstrated in **Figure 5B**. Meanwhile, the dosing period involved the measurement of body weights for every group of mice. The FA group mice did not show a significant weight increase, but the Cal/Bms group showed a trend of significant weight loss, as summarized in **Figure 5C**. In addition, H&E staining revealed that IMQ-induced psoriatic lesions, which included thickening of acanthosis cell layers, parakeratosis, and thickenings that extend down between dermal papillae (**Figure 6A**). Simultaneously, the epidermal and dermal layers of mice skin exhibited infiltration of inflammatory cells, accompanied by an augmentation in the count of macrophages, mast cells, and neutrophils (**Figure 6A**). The severity of skin injury in mice was not alleviated by the administration of LNs, AP. Nevertheless, the application of APLNs, Cal/Bms, and FA resulted in a substantial decrease in the thickening of acanthosis cell layers, alleviated epidermal parakeratosis, and diminished infiltration of inflammatory cells (**Figure 6A**). Findings indicate that positive drugs (Cal/Bms, FA) are effective in reducing psoriasis in IMQ-induced mice, yet one must consider possible side effects, such as a discernible pattern of weight reduction in the Cal/Bms group. Furthermore, APLNs are also capable of reducing psoriasis in IMQ-induced mice, verifying their specific anti-inflammatory and anti-psoriatic properties.

### 3.6 APLNs down-regulate serum inflammatory cytokines in IMQ-induced psoriasis in mice

We also evaluated a series of inflammation-related cytokines in mouse serum. The Multi-Analyte Flow Assay test showed that IMQ-induced psoriasis in mice had a notable effect on reducing IFN-γ, TNF-α, IL-9, IL-17F, IL-22, and IL-17A psoriasis-related inflammatory cytokines in the APLNs, Cal/Bms, and FA groups. However, solely IL-17F and IL-17A exhibited a decrease in expression within the AP group, as depicted in **Figure 6B**. Despite this, there was no decrease in the expression of other inflammatory cytokines associated with psoriasis, suggesting the instability of the α-pinene stock solution and the restricted therapeutic impact on models related to psoriasis. Concurrently, this demonstrates that APLNs used in treating IMQ-induced mouse psoriasis can mitigate skin psoriasis in mice through the diminished levels of cytokines associated with psoriasis, including IL-17F, IL-22, and IL-17A etc.

### 3.7 APLNs inhibited inflammatory protein expression and inflammatory cytokines released in skin of IMQ-Induced mouse model

**Figure 7:**
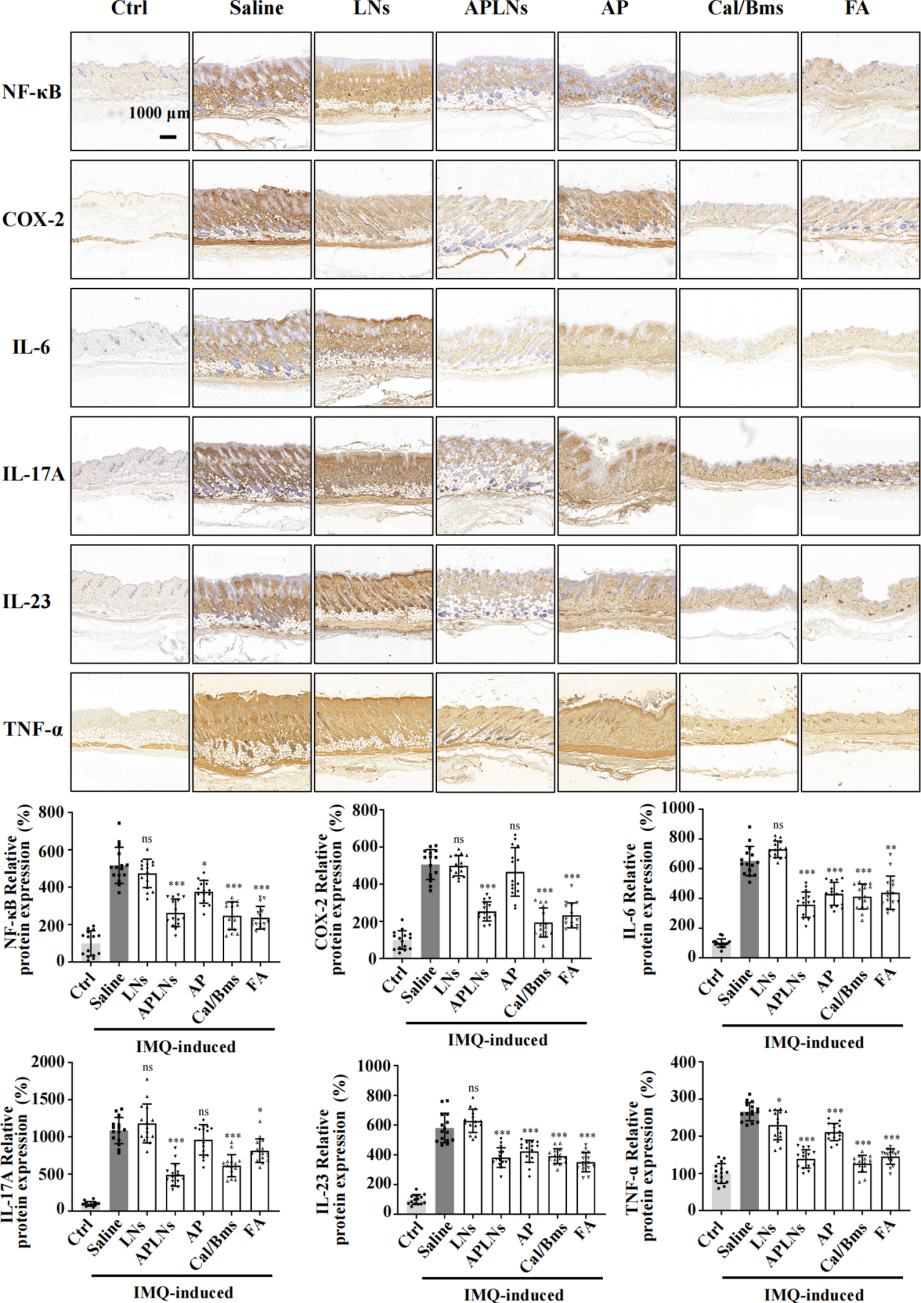
APLNs downregulate the expression of psoriasis-associated proteins and related cytokines through an IMQ-induced mouse psoriasis model. Immuno-histochemical staining of NF-κB, COX-2, IL-6, IL-17A, IL-23, and TNF-α in mouse skin tissues and relative intensity was counted. Scale bar 1000 μm. N = 5 mice for all groups. NS., not significant. ^*^, ^**^ and ^***^ represents *p* < 0.05, *p* < 0.01 and *p* < 0.001 comparing with the Saline.

An immunohistochemical staining of skin tissues was conducted to evaluate the influence of APLNs on the expression of inflammation-associated proteins and inflammatory cytokines in IMQ-induced mouse model skin. **Figure 7** illustrates that IMQ caused the NF-κB protein and inflammatory cytokines COX-2, TNF-α, IL-6, IL-17A, and IL-23 to be present in the dermis and epidermis when compared to the control group. Meanwhile, mice treated with APLNs, FA, and Cal/Bms exhibited a significant decrease in the expression of NF-κB protein, COX-2, TNF-α, IL-6, IL-17A, and IL-23 in comparison to the model group. In contrast, the AP group was not significantly down-regulated, and the LNs group did not trend to down-regulation, as shown in **Figure 7**. This further reveals the instability of the α-pinene stock solution, which complicates the generation of therapeutic effects. In summary, APLNs are capable of suppressing the secretion and expression of inflammatory cytokines associated with psoriasis by regulating NF-κB expression in IMQ-induced psoriasis in mice, thereby exerting their anti-psoriasis properties.

### 3.8 Effect of APLNs on spleen in mice with IMQ-induced psoriasis model

Research has suggested that mice afflicted with IMQ-induced dermatitis may experience an enlargement and weight gain of the spleen, as well as an alteration in its immune cell composition (39). Splenomegaly is a sign of an intensified systemic inflammatory response and is a key factor in the development of systemic inflammatory skin conditions, such as psoriasis (40). If the proportion of spleen to body wt% increases, then the spleen is populated with more immune cells, indicating a heightened immune response (40).

**Figure 8:**
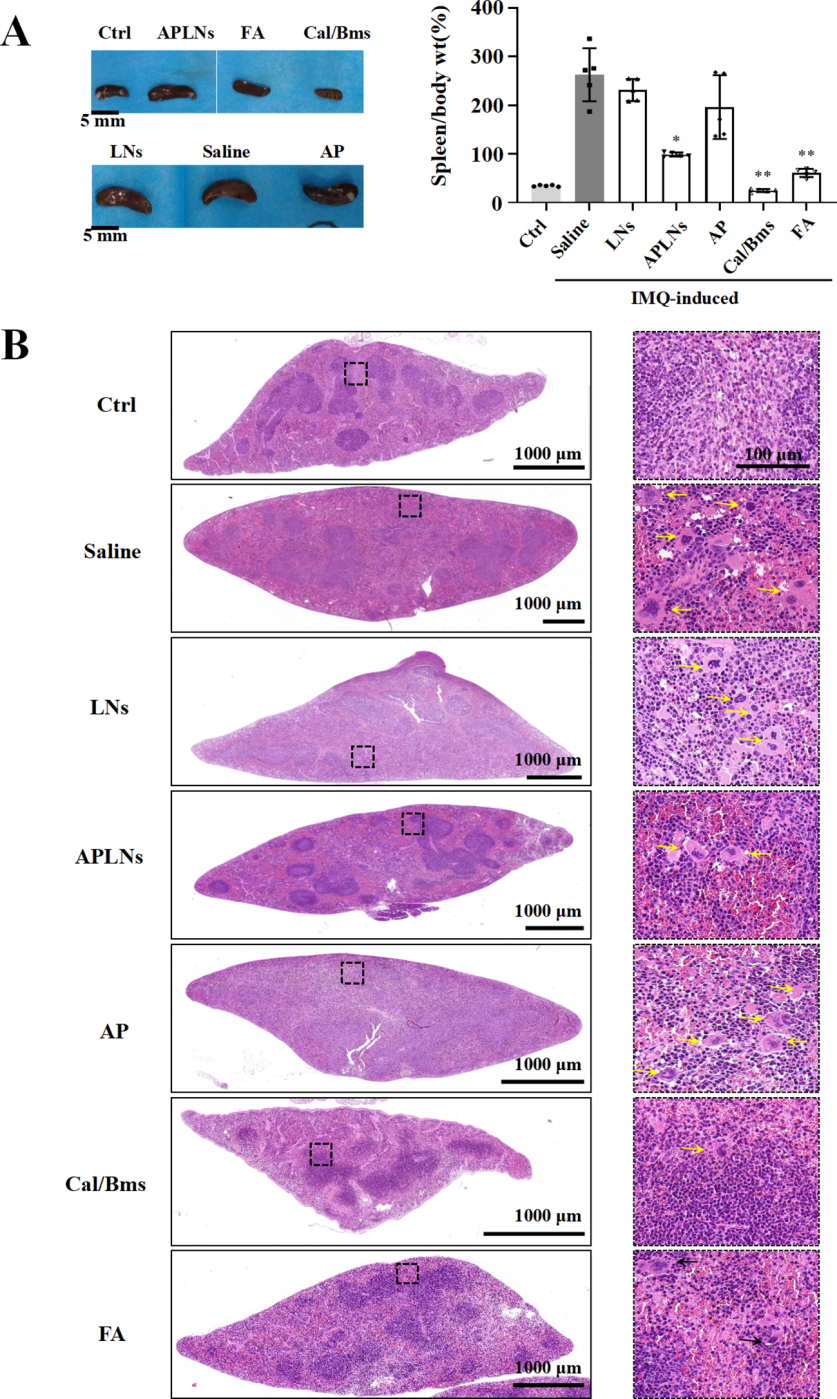
APLNs were able to alleviate spleen side effects of IMQ-induced psoriasis in mice. **(A)** Pictures of the spleens of mice in each group were taken and counted spleen/body weight percentage. Scale bar 5 mm. N = 5 mice for all groups. ^*^, ^**^ represents *p* < 0.05, *p* < 0.01 comparing with the Saline. **(B)** H&E staining mouse spleen and observation of the frequency of foreign body giant cells. Scale bar, 1000 μm for lower magnification and 100 μm for the higher.

Following an 8-day treatment, the IMQ model mice exhibited a significantly greater proportion of spleen/body weight compared to the control group, suggesting a rise in splenocyte count. Meanwhile, the APLNs, Cal/Bms, and FA groups exhibited a noteworthy decrease in spleen/body weight percentage when compared to the IMQ model group, as shown in **Figure 8A**. But, the body weight statistics of mice showed a decrease in body weight in the Cal/Bms group after administration, as well as an unchanged body weight increment in the FA group, as shown in **Figure 5C**. Betamethasone has been reported to inhibit body weight gain (41). Notably, APLNs did not impede the increase in mouse weight, as depicted in **Figure 5C**, according to body weight data. Additionally, the H&E staining revealed a lack of clarity in the structure between the white pulp and red pulp in the spleen of the IMQ model group, accompanied by a significant reduction in the area of the white pulp area in comparison to the control group, as demonstrated in **Figure 8B**. In the presence of proinflammatory signaling molecules, the spleen’s white pulp has the potential to serve as a site for immune response and may undergo growth (42). Nevertheless, APLNs, Cal/Bms, and FA groups were clearly in the structure between the white pulp and the red pulp, and there was no significant reduction in the area of the white pulp, as demonstrated in **Figure 8B**. Furthermore, within the IMQ model group, a substantial quantity of foreign body giant cells was generated through the aggregation of numerous macrophages in the red pulp area surrounding the white pulp of the spleen, occasionally manifesting in the dispersed white pulp area. However, the number of foreign body giant cells in APLNs, Cal/Bms, and FA groups was significantly lower than that in the IMQ model group, but it in APLNs was slightly more than that in Cal/Bms and FA groups, as demonstrated in **Figure 8B**. In total, the proportion of spleen/body weight notably decreased with the administration of APLNs, FA, and Cal/bms following IMQ induction. Meanwhile, the extent of spleen damage has diminished, yet the overall adverse effects of the positive drugs administered to the mice remain significant, such as weight loss and reduced spleen size. On the other hand, administering APLNs did not significantly cause systemic adverse effects in the mice.

## 4 Discussion and conclusions

Many conifer-derived essential oils contain α-pinene as their primary secondary metabolite. Numerous studies have documented a broad spectrum of its pharmacological activities (5). Due to the adverse effects of ethanol and certain organic solvents on the human body, as well as the instability of α-pinene, it proves challenging to develop a range of pharmaceutical dosage forms for medicinal purposes. In order to solve the above “shortcomings” of α-pinene, it was encapsulated into lipid nanoparticles and prepared as α-pinene lipid nanoparticles (APLNs). The pharmaceutical industry has seen the emergence of lipid nanoparticles (LNPs) as promising vehicles for the delivery of treatment agents including small molecules, nucleic acid drugs, etc, which have been extensively studied for their ability to transport both hydrophobic and hydrophilic molecules, such as small molecules, proteins, and nucleic acids (13). A number of nanocarriers, including liposomes (Doxil^®^, Lipusu^®^), nanoparticles (Abraxane^®^), and micelles (Genexol-PM^®^), had been approved for clinical cancer chemotherapy comparing with traditional formulations (43). Our study also observed that improved the stability and solubility of the fabricated APLNs. By testing, the average diameters and apparent zeta potentials of APLNs stored at 4°C for three months were approximately the same as those after fabrication, indicating that their stability was maintained for at least three months, as demonstrated in **Figure 1B**.

Administration of APLNs into the auricle of mice induced by xylene resulted in the alleviation of redness and swelling, suggesting the presence of its anti-inflammatory properties, as depicted in **Figures 4B–D**. Subsequently, we mainly assessed the anti-inflammatory ability of APLNs. The local aggregation of leukocytes, mast cells, and platelets, which secrete various lipid mediators (eicosanoids), proteins (cytokines and chemokines), and gaseous mediators (nitricoxide, carbon monoxide, reactive oxygen species) to circumscribe the affected site, trigger the immune response, eradicate the triggering factor, and thus reinstate physical health, is a multistep process currently acknowledged to be caused by inflammation (44). The activation of receptors influences various intracellular signaling pathways, such as MAPK, NF-κB, and JAK- STAT. The transcription factors stimulate the production of cytokines, influencing numerous inflammatory genes, including interleukins, interferons, Transforming Growth Factor (TGF), and chemokines (44). The application of APLNs resulted in a significant decrease in both mRNA levels of IL-6, IL-1β, iNOS, COX-2, TNF-α and protein levels of NF-κB, p-ERK, as shown in **Figures 2A, B**, when THP-1 cells were stimulated by LPS. RNA-sequencing and analysis also verified the above results, as depicted in **Figures 3A, B**. Therefore, it affirms the anti-inflammatory capacity of APLNs and their suppressive impact on proteins and cytokines associated with inflammation. NRF2 functions as a transcription factor, aiding cells in safeguarding against detrimental toxins and oxidative harm through the regulation of gene expression associated (37). Likewise, in THP-1 cells induced by LPS, there was a notable elevation in NRF2 protein levels subsequent to the administration of APLNs, following a dose-dependent manner. The decrease in KEAP-1 and increase in HO-1 also demonstrate that APLNs control pathways associated with oxidative stress response, as depicted in **Figure 2B**. Research has suggested that NRF2 can obstruct inflammation by directly restraining the transcription of proinflammatory cytokine genes or restraining the activity of inflammatory NF-κB signaling (45). It can be inferred from these results that APLNs have multiple pathways, including NRF2, NF-κB, MAPK, and potentially anti-inflammatory ones.

*Psoriasis* is a long-term, immune-related inflammatory skin disease that is linked to numerous illnesses and significantly reduces the quality of life for around 125 million people around the globe (28, 46). At present, topical treatments continue to be the fundamental approach in managing mild psoriasis. Patients with mild psoriasis may choose to receive a variety of treatments, including topical corticosteroids, targeted phototherapy, keratolytic agents, vitamin D analogs, and calcineurin inhibitors (28). In clinical practice, topical corticosteroids are typically used as the first line of treatment and are often combined with other drugs (47). The primary approach to treating mild or localized psoriasis for the majority is the administration of topical corticosteroids (47). The action of topical corticosteroids involves the down-regulation of genes that encode proinflammatory cytokines, leading to anti-inflammatory, antiproliferative, and locally vasoconstrictive effects (28). Nevertheless, the adverse reactions of topical corticosteroids have also been progressively identified through extensive clinical trials, mainly manifested as skin atrophy, telangiectasia, and striae (47). Minimizing the long-term usage of high-potency topical corticosteroids on extensive body surface areas, particularly in children, can mitigate rare systemic adverse effects like the suppression of the hypothalamus-pituitary and adrenal gland axis (29). In our experiments, we employed two corticosteroids: Cal/Bms and FA, as positive drugs, and the experimental results indicated that they could significantly relieve psoriasis after IMQ induction. However, the Cal/Bms group exhibited a substantial decrease in mouse body weight, as depicted in **Figure 5C**, which might be much worse to patients in clinical due to a prolonged application of these drugs. In the contrary, our APLNs could as well relief symptoms of psoriasis without possessing any observable side effects, especially for the loss of body weight.

Psoriatic lesions are caused by hyperproliferation, as well as disturbed in the differentiation of epidermal keratinocytes, which are triggered by immune mediators acting on the IL-23 and IL-17 pathways (23). The genetic connections highlight the potential participation of keratinocytes in the development of psoriasis, specifically the gene responsible for caspase recruitment domain-containing protein 14 (CARD14), which is associated with NF-κB signaling (23, 48). The experiment revealed that mice with IMQ-induced skin psoriasis were more likely to experience relief when APLNs were administered. The results of the IHC and serum Multi-Analyte Flow Assay indicated that APLNs had the ability to decrease the expression of IL-17, IL-23, TNF-α, and NF-κB, as demonstrated in **Figures 6B** and **7B**. This implies that our APLNs possess regulatory factors and protein pathways for the treatment of psoriasis. Furthermore, through the detection of serum and H&E staining of the spleen, it has been revealed that APLNs have the ability to regulate not only local skin lesions but also the systemic inflammatory response, as depicted in **Figures 6B** and **8B**.

**Figure 9:**
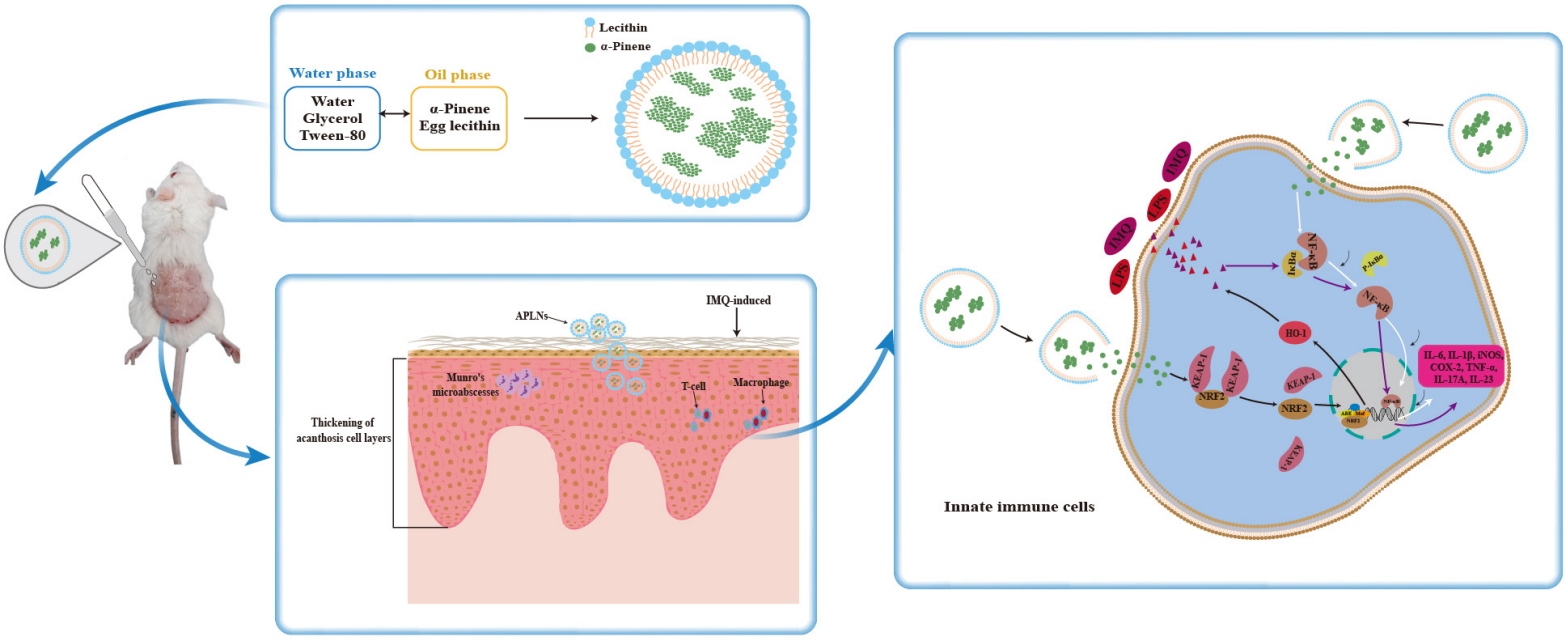
Fabrication of APLNs and its mechanism of diminished imiquimod-induced psoriasis-like skin injury.

In all, we successfully fabricated water soluble and aqueous stable APLNs, and studied its anti-inflammatory and anti-psoriasis effects both *in vitro* and *in vivo* as demonstrated in **Figure 9**. We sincerely believe that this APLNs could be of great potential for medical use in the future.

## Data Availability

The original contributions presented in the study are publicly available. This data can be found here: https://doi.org/10.5281/zenodo.13948806.

## References

[1] Feyaerts AF Luyten W Van Dijck P. Striking essential oil: tapping into a largely unexplored source for drug discovery. Sci Rep. (2020) 10:2867. doi: 10.1038/s41598-020-59332-5 32071337 PMC7028914

[2] Harvey AL Edrada-Ebel R Quinn RJ. The re-emergence of natural products for drug discovery in the genomics era. Nat Rev Drug Discovery. (2015) 14:111–29. doi: 10.1038/nrd4510 25614221

[3] Allenspach M Valder C Flamm D Grisoni F Steuer C. Verification of chromatographic profile of primary essential oil of pinus sylvestris L. Combined with chemometric analysis. Molecules. (2020) 25:2973–13. doi: 10.3390/molecules25132973 PMC7411901 32605289

[4] Vespermann KA Paulino BN Barcelos MC Pessôa MG Pastore GM Molina G. Biotransformation of α- and β-pinene into flavor compounds. Appl Microbiol Biotechnol. (2017) 101:1805–17. doi: 10.1007/s00253-016-8066-7 28105487

[5] Allenspach M Steuer C. [amp]]alpha;-Pinene: A never-ending story. Phytochemistry. (2021) 190:112857. doi: 10.1016/j.phytochem.2021.112857 34365295

[6] Yang C Hu DH Feng Y. Antibacterial activity and mode of action of the Artemisia capillaris essential oil and its constituents against respiratory tract infection-causing pathogens. Mol Med Rep. (2015) 11:2852–60. doi: 10.3892/mmr.2014.3103 25522803

[7] Nóbrega JR Silva DF Andrade Júnior FP Sousa PMS Figueiredo PTR Cordeiro LV. Antifungal action of α-pinene against Candida spp. isolated from patients with otomycosis and effects of its association with boric acid. Nat Prod Res. (2021) 35:6190–3. doi: 10.1080/14786419.2020.1837803 33094646

[8] Khoshnazar M Parvardeh S Bigdeli MR. Alpha-pinene exerts neuroprotective effects via anti-inflammatory and anti-apoptotic mechanisms in a rat model of focal cerebral ischemia-reperfusion. J Stroke Cerebrovasc Dis. (2020) 29:104977. doi: 10.1016/j 32689608

[9] Hou J Zhang Y Zhu Y Zhou B Ren C Liang S. [amp]]alpha;-pinene induces apoptotic cell death via caspase activation in human ovarian cancer cells. Med Sci Monit. (2019) 25:6631–8. doi: 10.12659/msm.916419 PMC6743669 31482864

[10] Kim DS Lee HJ Jeon YD Han YH Kee JY Kim HJ. Alpha-pinene exhibits anti-inflammatory activity through the suppression of MAPKs and the NF-κB pathway in mouse peritoneal macrophages. Am J Chin Med. (2015) 43:731–42. doi: 10.1142/s0192415x15500457 26119957

[11] Karthikeyan R Kanimozhi G Prasad NR Agilan B Ganesan M Srithar G. Alpha pinene modulates UVA-induced oxidative stress, DNA damage and apoptosis in human skin epidermal keratinocytes. Life Sci. (2018) 212:150–8. doi: 10.1016/j.lfs.2018.10.004 30292828

[12] Bouzenna H Hfaiedh N Giroux-Metges MA Elfeki A Talarmin H. Potential protective effects of alpha-pinene against cytotoxicity caused by aspirin in the IEC-6 cells. BioMed Pharmacother. (2017) 93:961–8. doi: 10.1016/j.biopha.2017.06.031 28724214

[13] Tenchov R Bird R Curtze AE Zhou Q. Lipid nanoparticles─From liposomes to mRNA vaccine delivery, a landscape of research diversity and advancement. ACS Nano. (2021) 15:16982–7015. doi: 10.1021/acsnano.1c04996 34181394

[14] Paliwal R Paliwal SR Kenwat R Kurmi BD Sahu MK. Solid lipid nanoparticles: a review on recent perspectives and patents. Expert Opin Ther Pat. (2020) 30:179–94. doi: 10.1080/13543776.2020.1720649 32003260

[15] Correia AC Monteiro AR Silva R Moreira JN Sousa Lobo JM Silva AC. Lipid nanoparticles strategies to modify pharmacokinetics of central nervous system targeting drugs: Crossing or circumventing the blood-brain barrier (BBB) to manage neurological disorders. Adv Drug Delivery Rev. (2022) 189:114485. doi: 10.1016/j.addr.2022.114485 35970274

[16] Liang WL Xiao L Gu HW Li XJ Li YS Zhang WK. Solid lipid nanoparticle induced apoptosis of macrophages via a mitochondrial-dependent pathway in *vitro* and in *vivo* . Int J Nanomedicine. (2019) 14:3283–95. doi: 10.2147/ijn.S200395 PMC6511261 31123400

[17] Medzhitov R. Origin and physiological roles of inflammation. Nature. (2008) 454:428–35. doi: 10.1038/nature07201 18650913

[18] Ostareck DH Ostareck-Lederer A. RNA-binding proteins in the control of LPS-induced macrophage response. Front Genet. (2019) 10:31. doi: 10.3389/fgene.2019.00031 30778370 PMC6369361

[19] Nestle FO Kaplan DH Barker J. Psoriasis. N Engl J Med. (2009) 361:496–509. doi: 10.1056/NEJMra0804595 19641206

[20] Boehncke WH Schön MP. Psoriasis. Lancet. (2015) 386:983–94. doi: 10.1016/s0140-6736(14)61909-7 26025581

[21] Rahman M Akhter S Ahmad J Ahmad MZ Beg S Ahmad FJ. Nanomedicine-based drug targeting for psoriasis: potentials and emerging trends in nanoscale pharmacotherapy. Expert Opin Drug Delivery. (2015) 12:635–52. doi: 10.1517/17425247.2015.982088 25439967

[22] Schön MP Boehncke WH. Psoriasis. N Engl J Med. (2005) 352:1899–912. doi: 10.1056/NEJMra041320 15872205

[23] Ghoreschi K Balato A Enerbäck C Sabat R. Therapeutics targeting the IL-23 and IL-17 pathway in psoriasis. Lancet. (2021) 397:754–66. doi: 10.1016/s0140-6736(21)00184-7 33515492

[24] Greb JE Goldminz AM Elder JT Lebwohl MG Gladman DD Wu JJ. Psoriasis. Nat Rev Dis Primers. (2016) 2:16082. doi: 10.1038/nrdp.2016.82 27883001

[25] Balato N Di Costanzo L Patruno C Patrì A Ayala F. Effect of weather and environmental factors on the clinical course of psoriasis. Occup Environ Med. (2013) 70:600. doi: 10.1136/oemed-2013-101505 23674841

[26] He X Zhu B Xie W He Y Song J Zhang Y. Amelioration of imiquimod-induced psoriasis-like dermatitis in mice by DSW therapy inspired hydrogel. Bioact Mater. (2021) 6:299–311. doi: 10.1016/j.bioactmat.2020.08.007 32954049 PMC7471623

[27] Coates LC FitzGerald O Helliwell PS Paul C. Psoriasis, psoriatic arthritis, and rheumatoid arthritis: Is all inflammation the same? Semin Arthritis Rheum. (2016) 46:291–304. doi: 10.1016/j.semarthrit.2016.05.012 27388027

[28] Armstrong AW Read C. Pathophysiology, clinical presentation, and treatment of psoriasis: A review. Jama. (2020) 323:1945–60. doi: 10.1001/jama.2020.4006 32427307

[29] Menter A Korman NJ Elmets CA Feldman SR Gelfand JM Gordon KB. Guidelines of care for the management of psoriasis and psoriatic arthritis. Section 3. Guidelines of care for the management and treatment of psoriasis with topical therapies. J Am Acad Dermatol. (2009) 60:643–59. doi: 10.1016/j.jaad.2008.12.032 19217694

[30] Lebwohl M Menter A Weiss J Clark SD Flores J Powers J. Calcitriol 3 microg/g ointment in the management of mild to moderate plaque type psoriasis: results from 2 placebo-controlled, multicenter, randomized double-blind, clinical studies. J Drugs Dermatol. (2007) 6:428–35. 17668541

[31] Soleymani T Hung T Soung J. The role of vitamin D in psoriasis: a review. Int J Dermatol. (2015) 54:383–92. doi: 10.1111/ijd.12790 25601579

[32] Oranje AP Marcoux D Svensson A Prendiville J Krafchik B Toole J. Topical calcipotriol in childhood psoriasis. J Am Acad Dermatol. (1997) 36:203–8. doi: 10.1016/s0190-9622(97)70281-0 9039169

[33] Zhou Y Zhou B Pache L Chang M Khodabakhshi AH Tanaseichuk O. Metascape provides a biologist-oriented resource for the analysis of systems-level datasets. Nat Commun. (2019) 10:1523. doi: 10.1038/s41467-019-09234-6 30944313 PMC6447622

[34] Veider F Akkuş-Dağdeviren ZB Knoll P Bernkop-Schnürch A. Design of nanostructured lipid carriers and solid lipid nanoparticles for enhanced cellular uptake. Int J Pharm. (2022) 624:122014. doi: 10.1016/j.ijpharm.2022.122014 35850184

[35] Danaei M Dehghankhold M Ataei S Hasanzadeh Davarani F Javanmard R Dokhani A. Impact of particle size and polydispersity index on the clinical applications of lipidic nanocarrier systems. Pharmaceutics. (2018) 10:57–2. doi: 10.3390/pharmaceutics10020057 PMC6027495 29783687

[36] Chen L Deng H Cui H Fang J Zuo Z Deng J. Inflammatory responses and inflammation-associated diseases in organs. Oncotarget. (2018) 9:7204–18. doi: 10.18632/oncotarget.23208 PMC5805548 29467962

[37] He F Ru X Wen T. NRF2, a transcription factor for stress response and beyond. Int J Mol Sci. (2020) 21:4777–13. doi: 10.3390/ijms21134777 PMC7369905 32640524

[38] Saha S Buttari B Panieri E Profumo E Saso L. An overview of Nrf2 signaling pathway and its role in inflammation. Molecules. (2020) 25:5474–22. doi: 10.3390/molecules25225474 PMC7700122 33238435

[39] Frenzel DF Borkner L Scheurmann J Singh K Scharffetter-Kochanek K Weiss JM. Osteopontin deficiency affects imiquimod-induced psoriasis-like murine skin inflammation and lymphocyte distribution in skin, draining lymph nodes and spleen. Exp Dermatol. (2015) 24:305–7. doi: 10.1111/exd.12649 25655893

[40] Park MY Choo YK Jeon SH Jang WG Lee JH Park JH. Therapeutic anti-psoriatic effects of myeloid-derived suppressor cells in combination with systemic tacrolimus (FK-506) in an imiquimod-induced mouse model of psoriasis. Int Immunopharmacol. (2020) 86:106553. doi: 10.1016/j.intimp.2020.106553 32563057

[41] Maekawa T Nojima H Kuraishi Y Aisaka K. The cannabinoid CB2 receptor inverse agonist JTE-907 suppresses spontaneous itch-associated responses of NC mice, a model of atopic dermatitis. Eur J Pharmacol. (2006) 542:179–83. doi: 10.1016/j.ejphar.2006.05.040 16824511

[42] Hjuler KF Gormsen LC Vendelbo MH Egeberg A Nielsen J Iversen L. Systemic inflammation and evidence of a cardio-splenic axis in patients with psoriasis. Acta Derm Venereol. (2018) 98:390–5. doi: 10.2340/00015555-2873 29327063

[43] Mu W Chu Q Liu Y Zhang N. A review on nano-based drug delivery system for cancer chemoimmunotherapy. Nanomicro Lett. (2020) 12:142. doi: 10.1007/s40820-020-00482-6 34138136 PMC7770879

[44] Fioranelli M Roccia MG Flavin D Cota L. Regulation of inflammatory reaction in health and disease. Int J Mol Sci. (2021) 22:5277–10. doi: 10.3390/ijms22105277 PMC8157220 34067872

[45] He F Antonucci L Karin M. NRF2 as a regulator of cell metabolism and inflammation in cancer. Carcinogenesis. (2020) 41:405–16. doi: 10.1093/carcin/bgaa039 PMC7298623 32347301

[46] Michalek IM Loring B John SM. A systematic review of worldwide epidemiology of psoriasis. J Eur Acad Dermatol Venereol. (2017) 31:205–12. doi: 10.1111/jdv.13854 27573025

[47] Patel RV Lebwohl M. In the clinic. Psoriasis. Ann Intern Med. (2011) 155:ITC2-1–ICT2-15. doi: 10.7326/0003-4819-155-3-201108020-01002 21810705

[48] Zhang XJ Huang W Yang S Sun LD Zhang FY Zhu QX. Psoriasis genome-wide association study identifies susceptibility variants within LCE gene cluster at 1q21. Nat Genet. (2009) 41:205–10. doi: 10.1038/ng.310 19169255

