# Supplementary figures and images for: Alpha-Pinene-encapsulated lipid nanoparticles diminished inflammatory responses in THP-1 cells and imiquimod-induced psoriasis-like skin injury and splenomegaly in mice
Source: Front Immunol. 2024 Oct 29;15:1390589. doi: 10.3389/fimmu.2024.1390589 (PMC11554515; doi:10.3389/fimmu.2024.1390589)

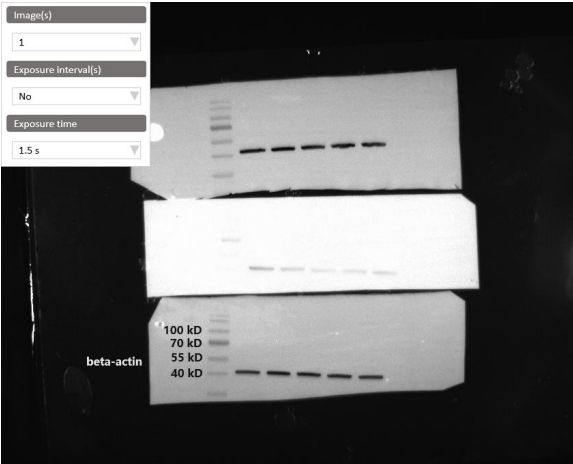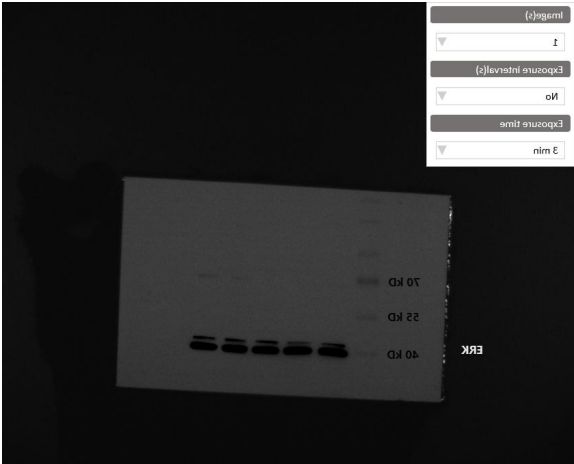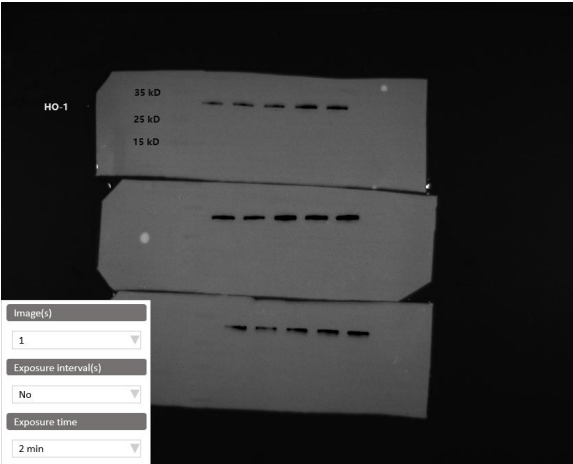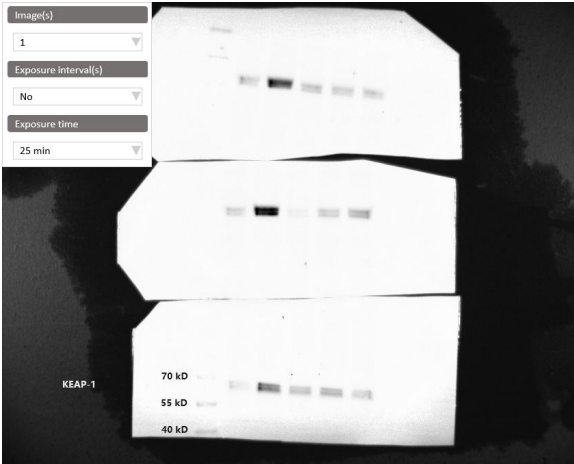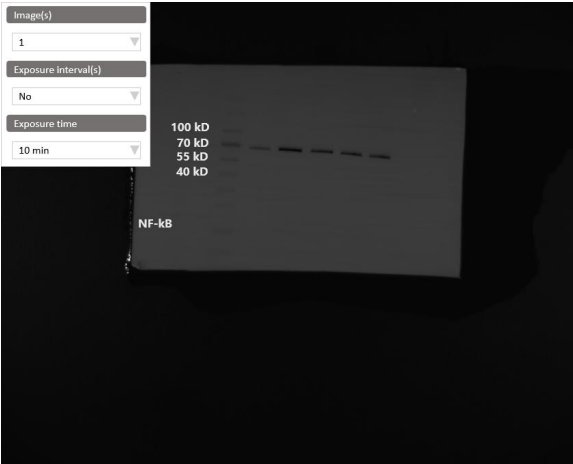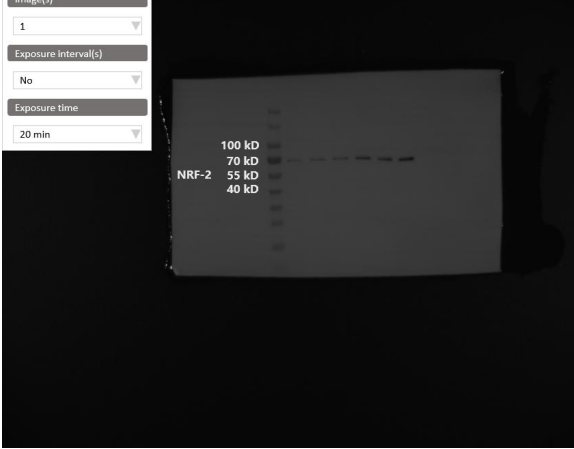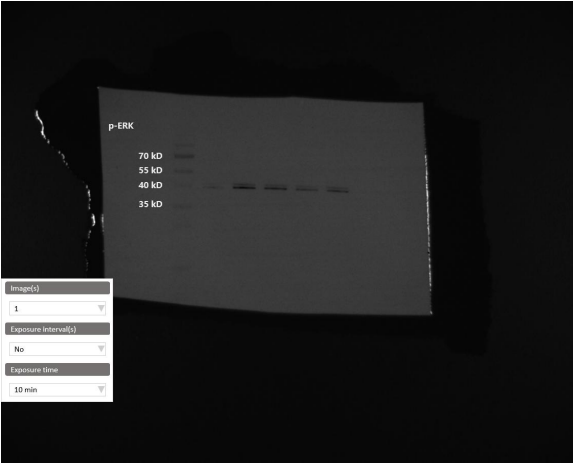

Supplement: Supplementary file 1 [file DataSheet1.pdf]
