# Supplementary material for: Alpha-Pinene-encapsulated lipid nanoparticles diminished inflammatory responses in THP-1 cells and imiquimod-induced psoriasis-like skin injury and splenomegaly in mice
Source: Front Immunol. 2024 Oct 29;15:1390589. doi: 10.3389/fimmu.2024.1390589 (PMC11554515; doi:10.3389/fimmu.2024.1390589)
Supplement: Supplementary file 2 [file DataSheet2.pdf]

Supplementary table S1. List of antibodies or reagents.

**Reagents or antibodies**	**Vendors**	**Country of vendors**	**Catalog numbers**	**Dilution Ratio**
(-)-α-Pinene	aladdin	USA	P110877	
Tween-80	MACKLIN	China	T818929	
Lecithin from egg yolk	yuanye	China	S31809	
Glycerol	Solarbio	China	#G8190	
Xylene	MACKLIN	China	X820585	
Acetonitrile	MACKLIN	China	E809064	
Isopropyl alcohol	MACKLIN	China	I811925	
Fetal bovine serum (FBS)	Gibco	USA	10270-106	
RPMI Medium Modified	Cytiva	USA	SH30809.01	
Cell Counting Kit-8(CCK-8)	YEASEN	China	40203ES80	
Lipopolysaccharides (LPS)	Sigma-Aldrich	USA	L4130	
Imiquimod Cream(IMQ)	MED-SHINE	China	NMPN: H20030128	
Calcipotriol-Betamethasone Ointment(Cal/Bms)	LEO Laboratories Limited	Denmark	IDL:H20160204	
Fluocinonide Cream	Tianjin Pacific Pharmaceutical	China	NMPN: H12020838	
RIPA Buffer	Thermo Fisher Scientific	USA	89900	
BCA protein quantification kit	CWBIO	China	CW0014S	
SDS gel electrophoresis	epizyme	China	PG213	
PVDF membranes	Merck Millipore	USA	ISEQ00010	
milk	BD Difco	USA	232100	0.5 g/ml
Tris-buffered saline	Bio Rad	USA	1706435	
Tween-20	Solarbio	China	T8220	
ECL	Bio Rad	USA	1705060	
GAPDH	CST	USA	#3683	WB 1:1000
NF-κB	CST	USA	#8242	WB 1:1000 IHC 1:500
HO-1	CST	USA	#26416	WB 1:1000
ERK	CST	USA	#4695	WB 1:1000
p-ERK	CST	USA	#4370	WB

				1:2000
COX2	CST	USA	#12282	IHC 1:1200
NRF2	CST	USA	#12721	WB 1:1000
KEAP1	CST	USA	#8047	WB 1:1000
HRP-linked goat anti-rabbit	CST	USA	#7074	WB 1:5000
horse anti-mouse IgG	CST	USA	#7076	WB 1:5000
IL-6	Abcam	UK	ab208113	IHC 1:100
IL-17A	Abcam	UK	ab79056	IHC 1:2000
IL-23	Abcam	UK	ab189300	IHC 1:2000
TNF alpha	Abcam	UK	ab1793	IHC 1:200

Supplementary table S2: Primer used for qPCR analysis

**Gene**	**Fw sequence**	**Rv sequence**
GAPDH	5'-GTCTCCTCTGACTTCAACAGCG-3'	5'-ACCACCCTGTTGCTGTAGCCAA-3'
iNOS	5'-GCTCTACACCTCCAATGTGACC-3'	5'-CTGCCGAGATTTGAGCCTCATG-3'
COX-2	5'-CGGTGAAACTCTGGCTAGACAG-3'	5'-GCAAACCGTAGATGCTCAGGGA-3'
IL-6	5'-AGACAGCCACTCACCTCTTCAG-3'	5'-TTCTGCCAGTGCCTCTTTGCTG-3'
IL-1β	5'-CCACAGACCTTCCAGGAGAATG-3'	5'-GTGCAGTTCAGTGATCGTACAGG-3'
TNF-α	5'-CTCTTCTGCCTGCTGCACTTTG-3'	5'-ATGGGCTACAGGCTTGTCACTC-3'
